# Security challenges and solutions using healthcare cloud computing

**DOI:** 10.25122/jml-2021-0100

**Published:** 2021

**Authors:** Mohammad Mehrtak, SeyedAhmad SeyedAlinaghi, Mehrzad MohsseniPour, Tayebeh Noori, Amirali Karimi, Ahmadreza Shamsabadi, Mohammad Heydari, Alireza Barzegary, Pegah Mirzapour, Mahdi Soleymanzadeh, Farzin Vahedi, Esmaeil Mehraeen, Omid Dadras

**Affiliations:** 1. School of Medicine and Allied Medical Sciences, Ardabil University of Medical Sciences, Ardabil, Iran; 2. Iranian Research Center for HIV/AIDS, Iranian Institute for Reduction of High Risk Behaviors, Tehran University of Medical Sciences, Tehran, Iran; 3. Department of Health Information Technology, Zabol University of Medical Sciences, Zabol, Iran; 4. School of medicine, Tehran University of Medical Sciences, Tehran, Iran; 5. Department of Health Information Technology, Esfarayen Faculty of Medical Sciences, Esfarayen, Iran; 6. Department of Health Information Technology, Khalkhal University of Medical Sciences, Khalkhal, Iran; 7. School of medicine, Islamic Azad University, Tehran, Iran; 8. Farabi Hospital, School of Medicine, Tehran University of Medical Sciences, Tehran, Iran; 9. Department of Global Health and Socioepidemiology, Graduate School of Medicine, Kyoto University, Kyoto, Japan

**Keywords:** health cloud, security, privacy, cloud computing, solutions, virtual network

## Abstract

Cloud computing is among the most beneficial solutions to digital problems. Security is one of the focal issues in cloud computing technology, and this study aims at investigating security issues of cloud computing and their probable solutions. A systematic review was performed using Scopus, Pubmed, Science Direct, and Web of Science databases. Once the title and abstract were evaluated, the quality of studies was assessed in order to choose the most relevant according to exclusion and inclusion criteria. Then, the full texts of studies selected were read thoroughly to extract the necessary results. According to the review, data security, availability, and integrity, as well as information confidentiality and network security, were the major challenges in cloud security. Further, data encryption, authentication, and classification, besides application programming interfaces (API), were security solutions to cloud infrastructure. Data encryption could be applied to store and retrieve data from the cloud in order to provide secure communication. Besides, several central challenges, which make the cloud security engineering process problematic, have been considered in this study.

## Introduction

Recently, clinical service demand on technology has been increased; cloud computing solutions, telemedicine, artificial intelligence, and electronic health can frequently provide better services [1]. Cloud computing is the delivery of different services through the Internet. These resources include tools and applications such as data storage, servers, databases, networking, and software [2]. Rather than owning their computing infrastructure or data centers, companies and organizations can lease access to whatever consists of storage or processing by the cloud service providers [3]. Shared resources, including servers, networks, storage tools, and application software, use cloud computing significantly [4, 5]. Also, cloud computing users can access their programs and information using the Internet as a conduit. The adoption of cloud technology has been increased in all industries, including healthcare [6, 7].

Healthcare organizations generate a wide range of data and information. Big data in the field of health need infrastructure for better storage and management. Patient data availability is one of the most vital needs in the health and medical industry [8]. Also, health researchers need easy access to extensive data for scientific analysis. Cloud technologies are applied in healthcare fields, such as mobile apps, patient portals, electronic medical records, devices with the Internet of Things (IoT), and big data analytics [9–11)] As per the service demands, healthcare providers need considerably to scale the data storage and network requirements.

Furthermore, using the cloud in electronic health records enables patients to easily and widely access their health information. Cloud computing changes how nurses, doctors, hospitals, and clinics deliver quality and profitable services to the patients. The challenges in the healthcare field include operational and infrastructure costs, security concerns to real-time information sharing, and robust backup.

Cloud computing has several advantages, including easy and convenient collaboration between users, reduced costs, increased speed, scalability, and flexibility. The data sharing process is more facilitated by cloud computing. Further, it has the potential to significantly decrease in-house infrastructural and operational costs in healthcare organizations [15]. By changing traditional data storage and handling procedures, cloud technology can speed up access to information and overcome the barriers that the industry stakeholders and patients encounter. Despite the numerous benefits of cloud computing, there are some drawbacks and challenges. Healthcare organizations are hesitant to adopt cloud computing due to security concerns, including patient information confidentiality, privacy, and service costs [16, 17]. Although massive data generated in healthcare organizations should be available to physicians and researchers, confidentiality concerns must be considered [18–20]. New challenges in cloud computing technology emerge in tandem with the growth, epiphany, and use of cloud technology in healthcare organizations; thus, identifying healthcare challenges and security issues appears essential. Accordingly, there are new challenges or security issues concerning offered solutions to cloud computing in healthcare organizations that should be examined and reviewed. Alongside identifying security challenges in cloud technology, reviewing present security solutions and providing new ones are also important objectives of this study. The present study aims at identifying barriers, challenges, security issues, and solutions in implementing cloud computing in the healthcare industry.

## Material and Methods

### Design

As this systematic review aimed at updating the results of earlier studies [21] concerning the topic, the eligible articles from the beginning of 2015 to November 2020 were retrieved. A comprehensive search of relevant literature was conducted utilizing the online databases of Scopus, Pubmed, Science Direct, and Web of Science. Two researchers were involved in the online search and identification of the relevant articles. In the first step, based on the inclusion criteria, the relevant studies were included using relevant keywords in the title or abstract. The literature not relevant to the present research and those with no original data were excluded. The second step involved a scrutinized full-text screening of the related article to choose the most eligible.

### Research question

This study aims to address the following issues of the modern healthcare systems:

• What are the main challenges threatening the security of cloud computing?
• What are the solutions to overcome these potential difficulties?

### Inclusion/Exclusion criteria

We included the English studies serving our study's purpose from the beginning of 2015 to November 2020.

The exclusion criteria were as follows:

• Preliminary data from incompleted projects;
• Abstracts, conference abstracts, and any other incomplete projects without full-text manuscripts;
• Review articles, letters to the editors, or any types of articles lacking original data.
• Articles lacking available free full-text.

## Results

**Figure 1.**
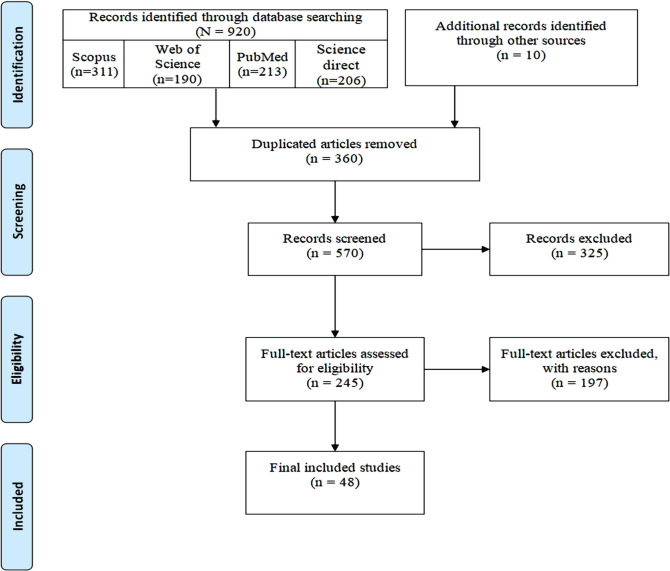
Flow diagram for the selection process of identified articles.

A total of 930 full texts of related studies were identified using the selected search strategy. After reviewing them, 360 duplicates were identified and removed; then, two independent investigators screened the title and abstract of the rest (570 resources). The full text of the extracted articles was reviewed, and the most relevant (245 resources) were selected based on the eligibility criteria. According to the selection criteria, 197 articles were excluded, all of which were found to be reviews (n=36), opinion articles (n=28), or not involving cloud security (n=133). Finally, 48 studies met inclusion criteria and were included in the final review (Figure 1).

**Figure 2.**
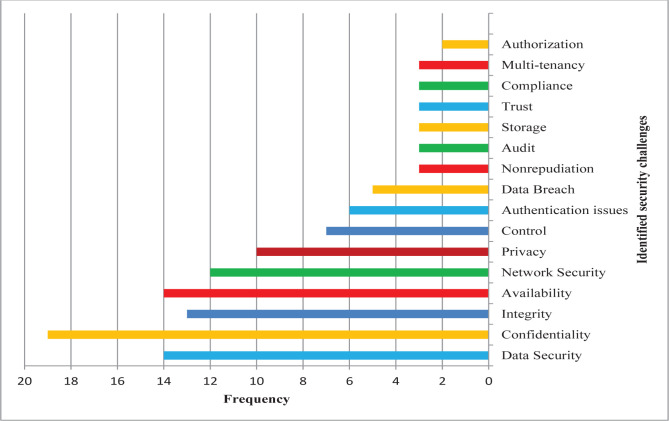
Frequency of the cloud computing security challenges.

**Figure 3.**
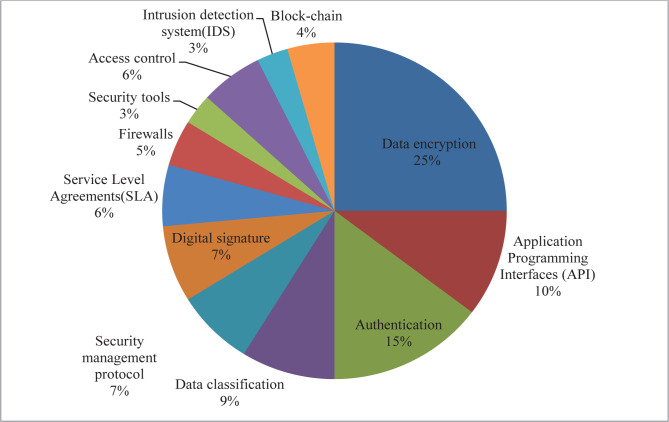
Most common identified solutions for security challenges in cloud computing.

We identified common security challenges and potential solutions for cloud technology. The reviewed studies and cloud computing security challenges and solutions are presented in Table 1. According to the review of the studies, the most frequent cloud security challenges were information confidentiality (n=19), data security (n=14), data availability (n=14), data integrity (n=13), and network security (n=12); frequency data are shown in Figure 2. Furthermore, data encryption (n=17), authentication (n=10), application programming interfaces (API)(n=7), and data classification (n=6) were the most common solutions for the security challenges in cloud infrastructure (Figure 3).

**Table 1.** Identified security challenges and potential solutions in healthcare cloud computing.

**ID**	**The first author (reference)**	**Publication date**	**Country**	**Study Context**	**Security challenges**	**Solutions recommended**
**1**	Dashti W [22]	2020	Pakistan	Security challenges in cloud computing	Availability, confidentiality, data integrity, control, audit, virtual machine security, network security	--
**2**	Ogiela L [23]	2020	Poland	Intelligent data management and security in cloud computing	Techniques of secret data management and protection	Cryptographic threshold techniques applied to split the secret in a specified group of trustees, being enhanced simultaneously using the shared secret intelligent linguistic threshold schemes
**3**	Tariq MI [24]	2020	Pakistan	Information security controls via Fuzzy AHP for cloud computing and wireless sensor networks	The proportionate security of networks	Fuzzy Analytical Hierarchy Process (FAHP); Analytical Hierarchy Process (AHP); Fuzzy AHP Methodology.
**4**	Tabrizchi H [20]	2020	Iran	Security challenges in cloud computing	Security policies, user-oriented security, application security, data storage, network	Data encryption (cryptography, quantum cryptography), secure sockets layer (SSL); Hash functions, message signature, message authentication code; Intrusion detection and prevention systems; firewalls, packet filters; Digital signature, endorsing certificate, notary; public and private blockchains
**5**	Wu B [25]	2020	China	Security and secure channel	Strategies to assure the confidentiality and security of outsourced sensitive data	Channel-free certificate less searchable public-key authenticated encryption (dCLPAEKS) scheme
****6****	Shakil KA[26]	2020	India	Healthcare management system	Identity theft, tax fraudulence, medical fraud, bank fraud, insurance fraud, and defamation of high-profile patients; Extending the capabilities of health applications over mobile devices, such as tablets, laptops, and smartphones.	Biometric-based authentication mechanism; BAMHealthCloud ensures the security of e-medical data access through a behavioral; Training of the signature samples for authentication purposes has been performed in parallel on the Hadoop MapReduce framework using Resilient Backpropagation neural; ALGO Health Security that performs security checks using parallelized MapReduce programming model.
**7**	George Amalarethinam DI [26]	2019	India	Cloud security challenges and solutions	Availability, confidentiality, privacy, integrity	Data encryption; OTP, digital certificate, and biometric verification; Rain-6 and digital signature.
**8**	Giri S [28]	2019	Nepal	Cloud security challenges and solutions	Data access and confidentiality	Data encryption and classification
**9**	Al-Issa Y [29]	2019	Jordan	Security challenges in eHealth cloud computing	Confidentiality, integrity, availability, ownership, and privacy of healthcare information; Authenticity, non-repudiation, audit, access control, data remanence and freshness, anonymity, unlinkability cloud multitenancy, secure transmission	HIPAA, HITECH ISO/IEC 27000-Series EU General Data Protection Regulation (GDPR) Patient-Centric Approach, Encryption Techniques
**10**	Bazm MM [30]	2019	France	Virtualization layer isolation challenges	Memory deduplication; large-page memory management, non-privileged access to hardware instructions.	Detection, countermeasure, application-level, OS-level, hypervisor-level, hardware-level, and moving target defense approaches
**11**	Modi KJ [31]	2019	India	Cloud security challenges and solutions	Security of data is a major factor, which restricts the acceptance of the cloud-based model.	Using linear network coding and re-encryption based on ElGamal cryptography in the form of a hybrid approach to secure healthcare information over the cloud; Linear network coding mechanism.
**12**	Kumar PR [32]	2018	Brunei	Cloud security challenges and solutions	Confidentiality, integrity, availability, authentication, authorization, non-repudiation	Creating the data, classifying the data, identifying the sensitive data, defining policies, and creating access methods for different data types; Creating policies for archiving and destroying data; Storing data with proper physical and logical security protection, including backup and recovery plan; Identifying which datatype can be shared, with whom and how it can be shared; defining data sharing policies; In cloud computing, many such policies are collectively called as Service Level Agreements (SLA); Creating a corrective action plan in case data is corrupted or hacked due to network or communication devices; security flaws while data is in transit; Data encryption; Using data duplication, redundancy, backups, and resilient systems to address availability issues.
**13**	Basu S [33]	2018	India	Security challenges in cloud computing	Confidentiality, integrity, availability	--
**14**	Pinheiro A [34]	2018	Brazil	Security architecture and protocol for trust verifications concerning the integrity of stored files in cloud services	Organizing a cloud storage service (CSS) that is safe from the client point of view, implementing CSS in public clouds, integrity, availability, privacy, and trust for the adopting cloud storage service	
**15**	Subramanian N [35]	2018	India	Security challenges in cloud computing	Cloud computing threats and risks, security in crypto-cloud	Infrastructure-as-a-Service, Platform-as-a-Service, Software-as-a-Service, Testing-as-a-Service, Security-as-a-Service, Database-as-a-Service
**16**	Stergiou C [36]	2018	Greece	Security, privacy, and efficiency of sustainable cloud computing for big data and IoT	The security and privacy	Installing a security "wall" between the cloud server and the Internet
**17**	Abrar H [37]	2018	Pakistan	Risk analysis of cloud sourcing in healthcare and public health industry	Data breaches, data loss, account hijacking, insecure interfaces and APIs, denial-of-service attacks (dos), malicious insiders, abuse of cloud resources, insufficient due diligence, shared technology issues	Likelihood determination, impact analysis, risk determination
**18**	Esposito C [38]	2018	Italy	Cloud security challenges and solutions	Confidentiality, privacy	Data encryption, blockchain
**19**	Huang Q [39]	2018	China	Data security challenges and solutions	Confidentiality, availability	Data encryption, public-key encryption, identity-based encryption, identity-based broadcast encryption, attribute-based encryption
**20**	Roy S [40]	2018	India	Cloud security challenges and solutions	The authentication process and security	A combined approach of fine-grained access control over cloud-based multi-server data along with a provably secure mobile user authentication mechanism for the Healthcare Industry 4.0.
**21**	Al-Shqeerat KH [41]	2017	Saudi Arabia	Security challenges in cloud computing	Network security, access control, cloud infrastructure, data security	Educating the stakeholders adequately on the cloud; Making sure that the IT administrator is able to control and manage cloud items and services when concluding the contract agreement with the service provider; An agreement with a third party to perform audits regularly to monitor the performance and compliance of the service provider to the agreed terms; Monitoring the performance of available cloud services and resources periodically; Data and applications in the cloud environment must be classified based on their values (according to their importance and sensitivity); not all data stored in the cloud are rated as top secure data; Backup and recovery; Proper authentication, authorization, and access security tools and mechanisms; Providing suite strong encryption protocols and key management for data at rest, in transit, and on the backup state
**22**	Barona R [42]	2017	India	Security challenges in cloud computing	Data breach, account or service traffic hijacking, insecure interfaces and Application Programming Interfaces (APIs), denial-of-Service (DOS), malicious insiders, abuse of cloud services, shared technology vulnerabilities	Information-centric security,high-assurance remote server attestation privacy-enhanced business intelligence, privacy and data protection, homomorphic encryption Searchable/ structured encryption, proofs of storage, server aided secure computation
**23**	Bhushan K [43]	2017	India	Security challenges in cloud computing	Physical level security issues, application and software-related security issues, network-related security issues, data-related security issues, computation-related issues, hardware virtualization-related issues, management and account control-related issues, trust-related issues, compliance and law-related issues	Classification based on the type technique used, classification based on the attack detection principle, classification based on reaction time, classification based on deployment point, classification based on the degree of deployment, classification based on the degree of cooperation, classification based on the defense activity, classification based on response strategy
**24**	Park J [44]	2017	Korea	Blockchain security in cloud computing	adapting blockchain security computing and its secure solutions	Blockchain provides security through the authentication of peers that share virtual cash, encryption, and generation of the hash value
**25**	Radwan T [45]	2017	Egypt	Cloud computing security	Privileged access, Data location; Availability, Investigation support; Regulatory compliance, Data segregation; Recovery, Long-term viability.	Authentication, authorization
**26**	Singh A [46]	2017	India	Cloud security issues	Zombie attack (DoS/DDoS attack); Service injection attack; Attack on virtualization/hypervisor; User to root attacks; Port scanning; Man-in-middle attack; Metadata spoofing attack; Phishing attack; Backdoor channel attack.	Strong authentication and authorization; Strong isolation mechanisms between VMs; Using the hash function to check service integrity; web service security; Adopting secure web browsers and API; Using a strong password; better authentication mechanism; Requiring strong port security, Requiring a proper Secure Socket Layer (SSL) architecture; Service functionality and other details should be kept in encrypted form to access the file required a strong authentication mechanism; Using a secure web link (HTTPS); Requiring strong authentication, authentication, and isolation mechanisms.
**27**	Mohit P [47]	2017	India	Cloud security challenges and solutions	Security protection is important for medical records (data) of the patients because of very sensitive information. Patient anonymity.	Authentication protocol for TMIS using the concept of cloud environment
**28**	Hussein NH [48]	2016	Sudan	Cloud security challenges and solutions	Authentication and authorization; Data confidentiality; Availability; Information security; Data access; Data breaches.	Logical network segmentation; Firewalls implementing; Traffic encryption; Network monitoring;
**29**	Kaur M [49]	2016	India	Cloud security challenges and solutions	Confidentiality; Authentication; Integrity; Non-repudiation; Availability	Data classification; Data Encryption
**30**	Muthurajan V [50]	2016	India	An elliptic curve-based Schnorrcloud security model in a distributed environment	The security upgrade in data transmission Approaches	A virtual machine-based cloud model with Hybrid Cloud Security Algorithm (HCSA); The combination of Elliptic Curve-based Schnorr (EC-Schnorr) scheme and blooming filter; A virtual machine-based cloud model with Hybrid Cloud Security Algorithm (HCSA); The optimization in the computational steps by ECC signature set and the duplication removal by blooming filter in the proposed Hybrid Cloud Security Algorithm (HCSA)
**31**	Prakash C [51]	2016	India	Cloud computing security	Deployment model issue, service model issues, network issues	Categorization of the data according to the risk associated with the data; Service Level Agreement (SLA); isolation amongst the resources by using segmentation; development of the dedicated application; a strong two-factor authentication
**32**	Vurukonda N [52]	2016	India	Privacy and confidentiality in the cloud environment	Data privacy and integrity, improper media sanitization, data recovery and vulnerability, data backup, service level agreements malicious insider, outside intruder, legal issues, confidentiality	SecCloud, for securing cloud data; FADE, a protocol for data privacy and integrity; TimePRE, a scheme for secure data sharing in the cloud; a methodology for security of resident data SPICE, identity management framework;role-based access control scheme, identity management framework
**33**	Alasmari S [53]	2016	USA	Security and privacy, challenges in IoT-based health cloud	Managing credentials and controlling access to applications and patient's confidential information, implementing and deploying cryptographic protocols in IoT health cloud correctly, mitigating device vulnerabilities and deploying firmware patches, securing IoT health networks and minimizing the risk of data loss	
**34**	Albuquerque SL [54]	2016	Brazil	Security in cloud-computing-based mobile health	Personal equipment vulnerabilities, assurance of cloud computing service availability assurance of confidentiality and integrity in unreliable cloud environments, access control and authenticity guarantee of systems users	
**35**	Casola V [55]	2016	Italy	Healthcare-related data in the cloud: challenges and opportunities	Having a decentralized and distributed design, allowing asynchronous interactions, supporting security mechanisms concerning privacy regulations, providing flexible data and service integration	Cryptographic solutions, such as privacy-preserving cloud ones
**36**	EL Bouchti A [56]	2016	Morocco	Cloud security challenges and solutions	Confidentiality, availability, data portability	Data encryption
**37**	Dorairaj SD [57]	2015	India	Cloud security challenges and solutions	Confidentiality, auditability	Access control, data encryption, integrity verification, log analysis, data classification
**38**	Kene SG [58]	2015	India	Cloud security challenges and solutions	Confidentiality, integrity availability	Hybrid detection technique, network intrusion detection system (NIDS)
**39**	Liu Y [59]	2015	USA	Cloud security challenges and solutions	Loss of control, lack of transparency, multi-tenancy	Data encryption, access control
**40**	Ali M [60]	2015	USA	Security challenges in cloud computing	Communication, architectural contractual and legal mobile application security, authentication, user privacy, data security	Using virtual LANs, IPS, IDS, and firewalls as a combination to protect the data in transit; Using off-the-shelf technology; Using standard algorithms; The implementers should secure each virtualized OS in each guest VM; The VMs at rest should be encrypted; Third-party security technology should be applied to decrease dependency on the CSP; VM images at rest should be patched with the latest fixes as soon as required; Security's vulnerability assessment tools should cover the virtualized environment; Virtualization-aware security tools should be implemented and used in the cloud computing environment; The protection mechanism should be in place until VMs are patched. The risk and attack models should be continuously built and maintained; Regular penetration testing for web applications should be carried out; The secure software lifecycle and software architecture should be developed and maintained; The source of the attributes should be as close to the master one as possible; Open standard federations, such as SAML and OAuth, should be preferred if possible; Bi-directional trust should be ensured for secure relationship and transactions.
**41**	Anand P [61]	2015	USA	Security challenges in cloud computing	Traffic hijacking, data breaches, data loss, insecure APIs, denial-of-Service, abuse of cloud services, malicious insiders, shared technology issues	--
**42**	Rao RV [62]	2015	India	Data security challenges in cloud computing	Integrity, confidentiality breaches, segregation, storage, data center operation	Encryption, RSA signature,identity-based cryptography, data security RSA-based storage security technique, distributed access control architecture
**43**	Wang B [63]	2015	USA	DDoS attack protection	Network security	The SDN-based network management, DaMask
**44**	Wang Y [64]	2015	Japan	Fog computing: security and forensics	Trust issue due to dependency on CSP, preserving the integrity, decentralization of logs, absence of critical information in logs, logs in multiple tiers and layers, volatility of logs, dependency on CSP for logs, dependability on CSP for data acquisition, trust issues of cloud computing, multi-tenancy, the chain of custody	API provided by CSP for logs, the cloud management plan, robust SLA, global unity, virtual machine introspection, the trusted third party, continuous synchronization, TPM, data provenance in the cloud, isolating a cloud instance
**45**	Moosavi SR [65]	2015	Finland	Cloud security challenges and solutions	End-to-end security for healthcare IoT	Session resumption-based end-to-end security scheme for healthcare Internet of things (IoT), The projected scheme is realized by using a certificate-based DTLS handshake between end-users and smart gateways, besides applying the DTLS session resumption method.
**46**	Zhang K [66]	2015	USA	Cloud security challenges and solutions	Security and privacy	Privacy-preserving health data aggregation, secure health data access and processing, misbehavior detection for the health-oriented mobile social network application
**47**	Zhou J [67]	2015	China	Cloud security challenges and solutions	E-healthcare cloud computing systems	Traceable and revocable multi-authority attribute-based encryption named TR-MABE to achieve efficiently multi-level privacy preservation without introducing other special signatures, secret keys used to protect patient's identity and PHI
**48**	Khattak HAK [68]	2015	Pakistan	Security concerns of cloud-based healthcare systems	Confidentiality, integrity, availability, privacy	Access control, multi-cloud computing security

## Discussion

Although cloud computing, as a novel technology, provides patient data availability all across, it encounters critical challenges in meeting one of the health industry's most significant demands. In cloud computing, providing security systems is necessary due to its inherent features, such as remote data storage, lack of network environment, proliferation, and massive infrastructure sharing [69]. Therefore, accurate identification of security challenges and their appropriate solutions is essential for both cloud computing providers and organizations using this technology [62].

Recently, artificial intelligence (AI) has shown a promising bright future in medical issues, especially when combined with cloud computing. Ahmed Sedik *et al.* have used AI deep learning to create a tool for quick screening of COVID-19 patients from their chest X-rays. This modality can be performed through a cloud-based system anywhere radiography equipment is found [70].

Identification systems also can use cloud computing. Alsmirat *et al.* have shown that digital cameras can act as a fingerprint identification system with an image compression rate of 30–40%, widely available on smartphones. Data security is a significant challenge there as well [71].

The present study indicates that the most vital challenge in cloud computing technology is maintaining data security. Malicious or negligent individuals may threaten data security. Several solutions provide data security, the most important of which is data encryption [20, 25, 29]. Data encryption is an essential line of protection in cybersecurity architecture. Encryption makes interrupted data use as difficult as possible [27, 28, 32]. Furthermore, data encryption is used to develop an encryption scheme that hypothetically can merely be broken with large amounts of computing potency [41, 42, 44, 49]. Kaur *et al.* [49] and Dorairaj *et al.* [57] have stated data encryption as a strategy to protect data against security threats. The results of the current research further show data encryption as the best solution to provide data security.

Many methods have been proposed for data encryption. A four-image encryption scheme has been proposed by Yu *et al.* based on the computer-generated hologram, quaternion fresnel transforms (QFST), and two-dimensional (2D) logistic-adjusted-sine map (LASM). This innovative technology considerably decreases the key data sent to the receiver for decryption, making it more promising to be stored and transmitted [72]. In order to secure cloud data storage and its delivery to authorized users, a hierarchal identity-based cryptography method has been proposed by Kaushik *et al.* to assure that a malicious attacker or CSP does not change for its benefit [73].

Another research has proposed a method to avoid always using the upstream communication channel from the clients to the cloud server via an optimistic concurrency control protocol, which reduces communication delay for IoT users. Only update transactions are sent to the cloud using this method, and they are only partially validated at the fog node [74].

According to the present research results, confidentiality is the second most important challenge in cloud technology. It refers to the protection of data from being obtained by unauthorized individuals; in other words, sensitive information is only accessible by authorized persons [75]. Cloud data control can result in an increased risk of data compromise. To ensure that the patient-doctor relationship runs smoothly, patients must have faith in the healthcare system to keep their data private [17]. Studies have shown that confidentiality may be achieved by access control [52, 62] and authentication [45, 76]. A Mutual Authentication and Secret Key (MASK) establishment protocol has been presented by Masud *et al.* in the field of the Internet of Medical Things (IoMT) in COVID-19 patients. The proposed protocol uses Physical Unclonable Functions (PUF) to enable the network devices to validate the doctor legitimacy (user) and sensor node before establishing a session key. Therefore, it addresses the confidentiality, authentication, and integrity problems and secures the sensitive health information of the patients [77].

This research shows that integrity, availability, and network security are important issues in the cloud computing infrastructure. A developmental study has mentioned integrity and availability as challenging problems in implementing cloud-based services, especially when losing or leaking information could result in major legal- or business-related damage [34]. Confidentiality, Integrity, and Availability (CIA) have been reported as the main three factors in cloud system security, which are considered here for the evaluation [33].

The number of network security challenges has rapidly increased with the advent of wireless sensor networks [22, 24]. Therefore, network security in cloud infrastructure has become a challenge for organizations [41, 43]. The common network attacks have happened at the network layer, including IP spoofing, port scanning, man-in-middle attack, address resolution protocol (ARP) spoofing, routing information protocol (RIP) attack, denial of service (DoS), and distributed denial of service (DDoS) [58]. The attackers, for instance, can send a considerable number of requests in order to access virtual machines in cloud computing to restrict their availability to valid users; this is termed the DoS attack. The availability of cloud resources is targeted by this attack [63]. The related studies have shown that no specific security standard exists for security controls in wireless networks [24, 51, 63]. However, in order to keep security in cloud computing networks, potential solutions, including Application Programming Interfaces (API), data classification, and security management protocol, could be applied [60, 64, 78, 79].

### Limitations

Due to the nature of the solution protocols, we could not explain their details. We aimed to clarify the present challenges and possible solutions to help others address and work on the issues, thus skipping some details and protocols presented for solutions. We only reviewed the English studies, thus possibly missing some reports.

## Conclusion

Cloud computing offers various benefits in data access and storage, particularly to healthcare organizations and relevant studies. Although the cloud computing environment is considered as a potential Internet-based computing platform, the security concerns encountered are notable. Security concerns may occur as a result of the cloud computing paradigm's shared, virtualized, and public nature. Overcoming these challenges by developing novel solutions is the only option for cloud computing adoption. All users, individuals or organizations, should be well informed of the security risks in the cloud.

In this study, an overview of cloud computing is presented; also, its security challenges and solutions surfaced within the past five years are reviewed. In order to offer safe data access, data encryption can be utilized to store and retrieve data from the cloud. We have also gone through some of the major challenges that make cloud security engineering tough. Identifying these challenges is the first step to tackle them, and future studies need to provide more feasible solutions to fix such bugs.

## Acknowledgments

The present study was extracted from the research project with the IR.KHALUMS.REC.1400.001 code entitled "Investigating the necessary infrastructure for implementing cloud computing technology in Khalkhal University of Medical Sciences" conducted at the Khalkhal University of Medical Sciences in 2021.

### Conflict of interest

The authors declare that there is no conflict of interest.
